# Neonatal hypothermia and associated factors among neonates admitted to neonatal intensive care unit of public hospitals in Addis Ababa, Ethiopia

**DOI:** 10.1186/s12887-018-1238-0

**Published:** 2018-08-04

**Authors:** Birhanu Wondimeneh Demissie, Balcha Berhanu Abera, Tesfaye Yitna Chichiabellu, Feleke Hailemichael Astawesegn

**Affiliations:** 1 Department of Nursing, College of Health Sciences and Medicine, Wolaita Sodo University, Sodo, Ethiopia; 2 School of Nursing and Midwifery, College of Health Science, Addis Ababa University, Addis Ababa, Ethiopia; 3 School of Public Health, College of Medicine and Health Sciences, Hawassa University, Hawassa, Ethiopia

**Keywords:** Hypothermia, Newborn, NICU, Addis Ababa

## Abstract

**Background:**

Neonatal hypothermia is a worldwide problem and an important contributing factor for Neonatal morbidity and mortality especially in developing countries. High prevalence of hypothermia has been reported from countries with the highest burden of Neonatal mortality. So the aim of this study was to assess the prevalence of Neonatal hypothermia and associated factors among newborn admitted to Neonatal Intensive Care Unit of Public Hospitals in Addis Ababa.

**Methods:**

An institutional based cross-sectional study was conducted from March 30 to April 30, 2016, in Public Hospitals in Addis Ababa and based on admission rate a total of 356 Neonates with their mother paired were enrolled for the study. Axillary temperate of the newborn was measured by a digital thermometer at the point of admission. Multivariate binary logistic regression, with 95% confidence interval and a *p*-value < 0.05 was used to identify variables which had a significant association.

**Results:**

The prevalence of Neonatal hypothermia in the study area was 64%**.** Preterm delivery (AOR = 4.81, 95% CI: 2.67, 8.64), age of Neonate ≤24 h old (AOR = 2.26, 95% CI: 1.27, 4.03), no skin to skin contact with their mother immediately after delivery (AOR = 4.39, 95% CI: 2.38, 8.11), delayed initiation of breastfeeding (AOR = 3.72, 95% CI: 2.07, 6.65) and resuscitation at birth (AOR = 3.65, 95%CI: 1.52, 8.78) were significantly associated with hypothermia.

**Conclusions:**

The prevalence of Neonatal hypothermia in the study area was high. Preterm delivery, age ≤ 24 h old, no skin to skin contact immediately after delivery, delayed initiation of breastfeeding and resuscitation at birth were independent predictors of Neonatal hypothermia. Therefore attention is needed for thermal care of preterm newborn and use of low-cost thermal protection principles of warm chain especially on early initiation of breastfeeding, skin to skin contact immediately after delivery and warm resuscitation.

**Electronic supplementary material:**

The online version of this article (10.1186/s12887-018-1238-0) contains supplementary material, which is available to authorized users.

## Background

World Health Organization (WHO) defined Neonatal hypothermia as an axillary temperature less than 36.5 °c. Reduction of thermal stability has a long-term physiologic effect that leads to, death due to hypoxia, and hypotension [1]. Globally an estimated of four million newborns die within the first four weeks of life, which accounts 2/3rd of all deaths in the first year of life and 40% of under five deaths. Most Neonatal deaths (99%) arise in low and middle-income countries [2, 3]. In Ethiopia also there is high Neonatal mortality, 37 deaths per 1000 live birth [4].

Hypothermia is one of the important causes for Neonatal death and morbidity in developing countries, which increases mortality by five times, and recent studies showed that every 1 °c decrement of body temperature increases mortality by 80% [2, 5, 6]. The prevalence is high among countries with the highest burden of Neonatal mortality [7]. It is a problem of both home delivered (32 - 85%) and institutional delivery (11 to 90%) [8]. A study in Bangladesh reported 34% of Neonates had hypothermia out of NICU admission [9]. Reports in developing country show that greater than 90% of Neonates were hypothermic (temperature less than 36.5 °C) and 10.7% of the newborn were at less than 35.0 °C [10, 11]. In West African sub-region, a prevalence rate of 62% at the point of admission was reported [12]. In Ethiopia also there was a prevalence of hypothermia ranging from 53 to 69.8% [8, 13].

Prematurity is one of the risk factors for Neonatal hypothermia and it is the leading cause of Neonatal mortality which accounts 37% of Neonatal death in Ethiopia [4]. And the prevalence of preterm birth ranges from 10 - 25.9% [14, 15]. Both physical characteristics and environmental factors predispose the preterm infant to hypothermia [16].

In Ethiopia lack of adequate perinatal care is one of the factors for onset of hypothermia, there is a high prevalence of home delivery which accounts 73% and Institutional deliveries accounts only 26% [17]. Low socio-economic status, poor kangaroo mother care practice, low birth weight, bathing of a newborn within 24 h, delayed initiation of breastfeeding, a traditional practice of oil massage of Neonates and inadequate knowledge of thermal care among health workers are determinant factors for hypothermia [2, 18, 19].

Although hypothermia is rarely a direct cause of death, it contributes to Neonatal mortality as a comorbidity of severe Neonatal infections, preterm birth, and asphyxia [8]. Mortality rate was significantly higher among hypothermic babies (RR = 2.26, CI = 1.14–4.48).

Even though predisposing factors for hypothermia are easily preventable the problem of hypothermia remains an unanswered question and it is highly prevalent in developing nations including sub-Sahara Africa [2].

Ethiopia applies thermal care principle which is one of the components of essential newborn care (ENBC) recommended by WHO. Despite this intervention, the problem of hypothermia remains a challenge in Ethiopia [1, 20]. And the achievement of sustainable development goal (SDG) 3 of ensuring healthy lives and promote well-being for all at all age requires a remarkable reduction of Neonatal death. Even though reduction of Neonatal hypothermia contributes to the achievement of SDG 3, it sustains as a challenge [21].

Providing ENBC including thermal care or prevention of Neonatal hypothermia is one part of nursing care, but the problem of Neonatal hypothermia remains a worldwide problem, especially in sub-Saharan Africa. Therefore, the purpose of this study was to determine the prevalence of Neonatal hypothermia and associated factors among Neonates admitted to NICU of Public Hospitals in Addis Ababa. So, this study will provide baseline data on the prevalence of Neonatal hypothermia and identification of possible factors for the onset of Neonatal hypothermia in the area will have greater input to program managers and policy makers for designing, proper implementation and evaluation programs on reduction of Neonatal mortality and improvement of newborn care to achieve SDG 3. In addition, the study will help to improve quality of newborn care in the nursing profession, specifically thermal protection, by low - tech preventive measures and early detection and referral of hypothermia.

## Methods

### Study design and period

An institutional based cross -sectional study design was conducted from March 30 to April 30, 2016, to determine the prevalence of Neonatal hypothermia and associated factors among Neonates admitted to Neonatal Intensive Care Unit of Public Hospitals in Addis Ababa.

### Study setting

The study was conducted in six Public Hospitals in Addis Ababa, Ethiopia, that have their own NICU; namely; Tikur Anbessa Specialized Teaching Hospital that has its own Neonatal Intensive Care Unit (NICU) with an average NICU admission of 240 Neonates per month, St. Paul’s Hospital Millennium Medical College with an average NICU admission of 210 Neonates per month, Yekatit 12 Hospital Medical College with an average NICU admission of 170 Neonates per month, Gandhi Memorial Specialized Hospital with an average NICU admission of 192 Neonates per month, Zewditu Memorial Hospital with an average NICU admission of 110 Neonates per month and Tirunesh Beijing General Hospital with an average NICU admission of 60 Neonates per month. The study was conducted in all Public Hospitals in Addis Ababa that has their own NICU, because the level of perinatal care given, standards of NICU, and accessibility of thermal prevention materials are somewhat different in each Hospital.

### Population

#### Source population

The source populations were all Neonates who were admitted to NICU of public Hospitals in Addis Ababa.

#### Study population

Randomly Selected Neonates admitted to NICU of public Hospitals in Addis Ababa from March 30 to April 30, 2016, were the study population.

### Eligibility criteria

#### Inclusion criteria

All Neonates with their mother admitted to NICU of Public Hospitals in Addis Ababa during the study period were included in the study.

#### Sample size determination

Sample size was calculated by using single population proportion formula:

$$ \mathrm{n}=\frac{\kern0.50em \left(\mathrm{z}a/2\right){2}^{\ast }\ \mathrm{pq}}{d2} $$

By considering 10% none response rate of participants, the final sample size was **356**.

Where *n* = the required sample size.

$$ {\displaystyle \begin{array}{l}d=m\arg in\ of\ error\ between\ the\ sample\ and\ population=5\%=0.05\\ {}Z=s\tan dard\ normal\ distribution\ value\  at\ 95\% confidence\ level\\ {}Z\ \alpha /2=1.96\  for\ 95\% confidence \operatorname {int} erval\\ {}p=\Pr evalence\ of\ Neonatal\ hypothermia\ \left(69.8\%\right)\end{array}} $$

from the previous study conducted in Gondar University Teaching and Referral Hospital, Northwest Ethiopia [13].

### Sampling technique and procedure

There were a total of six Public Hospitals in Addis Ababa that have their own organized NICU and they have a total average number of 982 admissions to NICU per month and a total sample size of 356 Neonates were selected from the six Hospitals. Then participants was selected by using systematic random sampling technique, that is every three admission until the required sample size was obtained (K = 2.75, approximately every 3 admissions was taken). The number of Neonates surveyed from each Hospital was allocated proportionally to the total average number of admission per month from all Hospitals.

### Method of data collection

The instrument for data collection was semi-structured pre-tested questionnaire which was adopted and modified from a study conducted in Ethiopia, Gondar University Hospital, Nigeria and Uganda [12, 13, 19]. The questionnaire contains items to assess the temperature of the newborn during admission to NICU and associated factors for the onset of hypothermia (Additional file 1).

Axillary temperate of the newborn was measured for three minute by using digital thermometer (model of MT-101 MT-111) which can measure from 32.0 ^°^C to 42.9 °C (89.6 °F to 109.9 °F) that had measurement accuracy of ±0.1 °C for the temperature range of (35.5 °C – 42.0 ^°^C) and ± 0.2 °C for the temperature range of (32.0 ^°^C - 35.5 ^°^C or above 42.0 °C) at point of admission. The thermometer was disinfected by using 70% ethyl alcohol disinfectant with a damp cloth after every measure of axillary temperature of the newborn to prevent infection transmission.

And other data such as; medical diagnosis, and CPR history was collected from the chart of the newborn and socio-demographic data and obstetric history was collected from their mother by using semi-structured pre-tested questionnaire. Infrared thermometer (model of Kintrex IRT0421) with a measurement range of (− 60 °C to 50 °C) and measurement accuracy of ±2 ^°^C was used to measure the room temperature of the NICU. And data collection was done carefully by six BSc nurses.

### Study variables

#### Dependent variable

- Neonatal hypothermia

#### Independent variables

1. Socio-demographic characteristics of the mother
  Maternal age, parity, residence, ethnicity, educational status, occupation and income.
2. Neonatal, obstetric and environmental factors of the neonate:

Age of newborn in hour, sex of newborn, low birth weight, mode of delivery, pregnancy type (single / multiple), prematurity, skin to skin contact with mother immediately after delivery, bathing before age of 24 h, CPR, delayed initiation of breastfeeding, room temperature of NICU, place of delivery, application of oil massage, obstetric complication during pregnancy and Medical diagnosis during admission.

### Operational definitions

- **Hypothermia**: an axillary temperature of less than 36.5 °c
- **Cold stress(mild hypothermia)**: an axillary temperature of 36.0 to 36.4 °C
- **Moderate hypothermia**: an axillary temperature of 32.0 to 35.9 °C
- **Severe hypothermia:** an axillary temperature of < 32.0 °C
- **Normothermic:** an axillary temperature of 36.5 to 37.5 °C
- **Hyperthermia:** an axillary temperature of > 37.5 °C
- **Admission temperature**: The first temperature obtained from neonates at admission to NICU
- **Inborn**: a new born that was delivered from the study Hospital
- **Out born:** a new born that was deliver other than the study Hospital

### Data quality and control

The questionnaire was prepared in English and translated to Amharic, and back-translated into English by two language experts to check for consistency of the questionnaire. The data was collected by six BSc. nurse experts. Thermometer calibration was done for the reliability of the thermometer before using the instrument for data collection. Three day training and clear orientation were provided on the process of data collection for data collectors. A pretest was done by 5% of the study population in another Hospital three weeks before the actual data collection to evaluate the clarity of questions and validity of the instrument and reaction of respondents to the questions. Data collectors were closely monitored and guided by two MSc. nurse supervisors during data collection.

### Data entry and analysis

The data was cleaned manually, coded and entered into Epi info version 3.5 and exported to SPSS version 20 software for further analysis. After coding, and entering the data to the software descriptive statistics were used to calculate the result in proportion, frequencies, cross tabulation, and measure of central tendency. Tables and graphs were used to present the result. A bivariate binary logistic regression was used to identify candidate variables for the final model (multivariate binary logistic regressions) at *p* - value < 0.20. Finally the independent predictors or variables which had significant association were identified by using multivariate binary logistic regressions. The cut point to declare the presence of an association between the dependent and independent variable was *p* – value < 0.05 or AOR, 95% CI.

## Results

### Socio - demographic characteristics

A total of 356 mothers with their neonates were included in the study with 100% response rate. The mean age of mothers was 28 years (SD = 5.6) and more than half of the mothers were in the age group between 20 and 29 (51.1%) years of age. One hundred twenty seven (35.7%) were Oromo in ethnicity and majority of the mothers 206 (57.9%) were Orthodox followers. Two hundred seventy six (77.5%) were urban residents. Eighty respondents (22.2%) were unable to read and write and 144 (40.4%) of respondents were housewife. The mean monthly income of the family was 54 US dollar (SD = 11US dollar) and 117 (32.9%) had a monthly income of below average. And 191 respondents (53.7%) were primiparous (Table 1).

**Table 1:** Socio-demographic characteristics of mothers of neonates admitted to Neonatal Intensive Care Unit of Public Hospitals in Addis Ababa, Ethiopia, 2016 [*n* = 356]

Variables	Categories	Frequency	Percentage (%)
Age of mother (years)	15–19	17	4.8
	20–29	182	51.1
	30–39	145	40.7
	40–49	12	3.4
Ethnicity	Amhara	121	34.0
	Tigre	55	15.4
	Oromo	127	35.7
	Gurage	37	10.4
	Other	16	4.5
Religion	Orthodox	206	57.9
	Protestant	59	16.6
	Muslim	88	24.7
	Other	3	0.8
Residence	Urban	276	77.5
	Rural	80	22.5
Educational status	Unable to read and write	80	22.5
	Primary school	77	21.6
	Secondary school	102	28.7
	Diploma and above	97	27.2
Occupation	House wife	144	40.4
	Government employ	79	22.2
	Private business	92	25.8
	Student	27	7.6
	Farmer	14	3.9
Monthly income of the family	Below average	117	32.9
	Average (43–65 US dollar)	129	36.2
	Above average	110	30.9
Parity	Primiparous	191	53.7
	Multiparous	165	46.3

### Neonatal factors

Majority of Neonates were males 204 (57.3%) and the median age of the newborn was 3 h. And most of the neonates 233 (65.4%) were in the age group of ≤24 h. The mean birth weight was 2440 g (SD 721 g). More than half 183 (51.4%) of the Neonates had birth weight ≥ 2500 g. The mean gestational age (GA) was 36 weeks ±2.8 weeks, most of them, 202 (56.7%) were with GA < 37 weeks. Only 126 (35.4%) of Neonates had early initiation of breastfeeding within one hour after birth. Eighty four (23.6%) had received resuscitation (CPR) during birth (Table 2).

**Table 2:** Neonatal characteristics of respondents among Neonates admitted to Neonatal Intensive Care Unit of Public Hospitals in Addis Ababa, Ethiopia, 2016 [*n* = 356]

Variables	Categories	Frequency	Percentage (%)
Age of Newborn (hour)	≤24	233	65.4
	24–72	60	16.9
	> 72	63	17.7
Sex of new born	Male	204	57.3
	Female	152	42.7
Birth weight(grams)	< 1000	10	2.8
	1000–1499	32	9.0
	1500–2499	131	36.8
	2500–4000	179	50.3
	> 4000	4	1.1
Gestational age (weeks)	< 28 weeks	2	0.6
	28- < 32 weeks	25	7.0
	32- < 37 weeks	175	49.2
	37-42 weeks	152	42.7
	> 42 weeks	2	.6
Started breast feeding within one hour after birth	Yes	126	35.4
	No	230	64.6
Received CPR during birth	Yes	84	23.6
	No	272	76.4

### Obstetric and environmental factors

Most of the pregnancies 311 (87.4%) were single and the majority of Neonates 286 (80.3%) were born without any obstetric complication. More than half 213 (59.8%) were delivered through SVD. Sixty five (18.3%) of the newborn were bathed before 24 h old and more than half of Neonates 188 (52.8%) had no skin to skin contact immediately after birth. And 41 (11.5%) had Oil massage of the skin after birth. One hundred seventy (47.8%) were out born neonates and of them, nine (2.5%) delivered at home. More than half 190 (53.4%) deliver during day time. Majority of Neonates 329 (92.4%) were admitted to NICU at room Temperature ≥ 25 ^°^C (Table 3).

**Table 3:** Obstetric and Environmental characteristics of respondents among Neonates admitted to Neonatal Intensive Care Unit of Public Hospitals in Addis Ababa, Ethiopia, 2016 [*n* = 356]

Variables	Categories	Frequency	Percentage (%)
Obstetric complication during pregnancy	Yes	70	19.7
	No	286	80.3
pregnancy type	Single	311	87.4
	Twine	41	11.5
	Triple	4	1.1
Mode of delivery	SVD	213	59.8
	Instrumental	32	9.0
	C/S	111	31.2
skin to skin contact immediately after delivery	Yes	168	47.2
	No	188	52.8
Place of delivery	Inborn	186	52.2
	Out born	170	47.8
setting for out born delivery	Missing (Inborn)	186	52.2
	Other Hospital	69	19.4
	Health Centre	76	21.3
	Private health facility	13	3.7
	Traditional birth center	3	0.8
	Homes	9	2.5
Oil massage of the skin immediately after birth	Yes	41	11.5
	No	315	88.5
Bathed the new born before 24 h old	Yes	65	18.3
	No	291	81.7
Time of delivery	Day time	190	53.4
	Night time	166	46.6
Room Temperature of NICU	< 25 ^°^C	27	7.6

### Medical diagnosis of the neonate

Medical diagnoses during admission were reviewed from medical record of the newborn and 116 (32.6%) were admitted for the reason of respiratory distress, 173 (48.6%) diagnosed as low birth weight and 202 (56.7%) were diagnosed as preterm, and 84 (23.6%) diagnoses as perinatal asphyxia (Table 4).

**Table 4:** Medical diagnoses of neonates during admission among Neonates admitted to Neonatal Intensive Care Unit of Public Hospitals in Addis Ababa, Ethiopia, 2016 [*n* = 356]

Variable	Categories	Frequency	Percentage (%)
Diagnosis during Admission	Respiratory distress	116	32.6
	Preterm	202	56.7
	Jaundice	55	15.4
	Sepsis	83	23.3
	LBW	173	48.6
	Perinatal asphyxia	84	23.6
	Congenital anomaly	35	9.8
	Meconium aspiration syndrome	22	6.2
	Small for gestational age	15	4.2
	hypoglycemia	15	4.2
	Other	16	4.5
	The total cumulative frequency for diagnosis is greater than 100% because the Neonate may have more than one clinical diagnosis during admission.		

### The prevalence of neonatal hypothermia

**Fig. 1:**
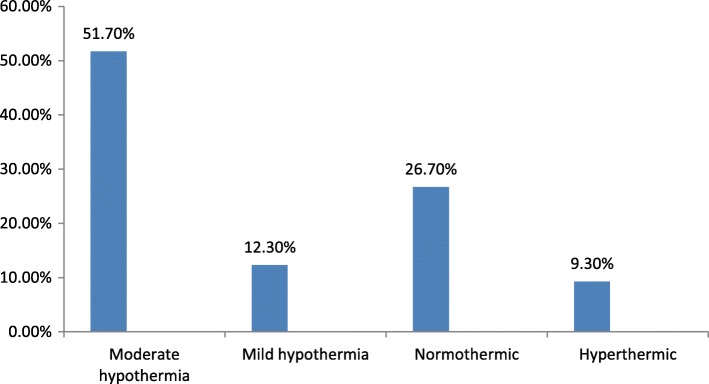
Classification of temperature among Neonates admitted to Neonatal Intensive Care Unit of Public Hospitals in Addis Ababa, Ethiopia, 2016 [*n* = 356]

The prevalence of neonatal hypothermia among Neonates admitted to Neonatal Intensive Care Unit of Public Hospitals in Addis Ababa was 228 (64%). Among them, more than half 184 (80.7%) were moderate hypothermic and the remaining 44 (19.3%) were mild hypothermic babies (Fig. 1).

**Fig. 2:**
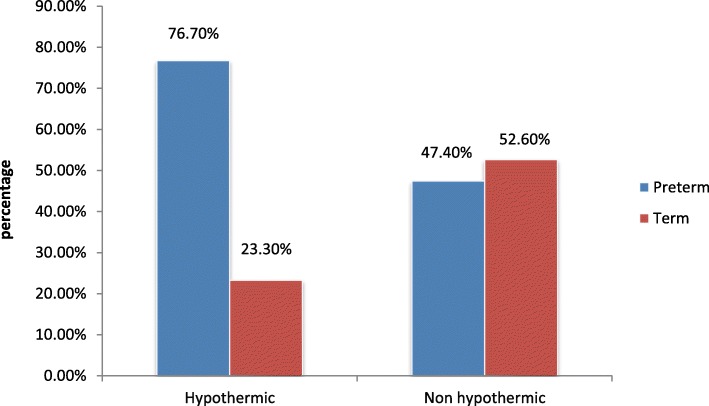
Comparison of Hypothermia with gestational age among Neonates admitted to Neonatal Intensive Care Unit of Public Hospitals in Addis Ababa, Ethiopia, 2016 [*n* = 356]

And the prevalence of hypothermia was high among preterm 155 (76.7%), low birth weight 127 (73.4%), age ≤ 24 h 171 (73.4%), and among out born delivery 112 (65.9%) (Fig. 2).

### Factors associated with neonatal hypothermia

In bivariate logistic regression analysis the following factors were significantly associated with hypothermia; age of newborn ≤24 h old, low birth weight, preterm delivery, no skin to skin contact to their mother immediately after delivery, no early initiation of breastfeeding within one hour, resuscitation at birth (CPR), obstetric complication during pregnancy, multiple Pregnancy and night-time delivery. Then those variables which are significant on bivariate analysis were entered to multiple logistic regressions to see independent predictors.

Accordingly, Neonates with the age of ≤24 h old were 2 times more likely to have hypothermia when compared to age greater than 24 h (AOR = 2.26, 95% CI: 1.27, 4.03).

Preterm Neonates were 4.8 times more likely to have hypothermia when compared to term delivery (AOR = 4.81, 95% CI: 2.67, 8.64). And newborn who had no skin to skin contact to their mother immediately after delivery were 4.3 times more likely to be hypothermic when compared to those who have skin to skin contact (AOR = 4.39, 95% CI: 2.38, 8.11). Those Neonates who had no early initiation of breastfeeding within one hour after birth were 3.7 times more likely to develop hypothermia when compared to those who have started within one hour after birth (AOR = 3.72, 95% CI: 2.07, 6.65). And Neonates who had resuscitation at birth (CPR) were 3.6 times more likely to be hypothermic when compared to those who had no resuscitation (AOR = 3.65, 95% CI: 1.52, 8.78) (Table 5).

**Table 5:** Bivariate and multivariate logistic regression analysis of associated factors among Neonates admitted to Neonatal Intensive Care Unit of Governmental Hospitals in Addis Ababa, Ethiopia, 2016 [*n* = 356]

Variables	Hypothermic (228)	Non Hypothermic (128)	COR (95% CI)	AOR (95% CI)	*P* - value
	*N* (%)	*N* (%)			
Age of Neonate (hour)					
≤ 24	171(73.4)	62(26.6)	3.19(2.02,5.05)	2.26(1.27, 4.03)	.005*
> 24	57(46.3)	66(53.7)	1.0	1.0	
Birth weight (grams)					
< 2500	127(73.4)	46(26.6)	2.24(1.44,3.5)	1.33(0.75,2.36)	0.331
≥ 2500	101(55.2)	82(44.8)	1.0	1.0	
Gestational age (weeks)					
< 37	155(76.7)	47(23.3)	3.66(2.32,5.76)	4.81(2.67, 8.64)	0.001*
≥ 37	73(47.4)	81(52.6)	1.0	1.0	
skin to skin contact					
Yes	71(42.3)	97(57.7)	1.0	1.0	0.001*
No	157(83.5)	31(16.5)	6.92(4.23,11.32)	4.39(2.38, 8.11)	
Early initiation of breast feeding					
Yes	45(35.7)	81(64.3)	1.0	1.0	0.001*
No	183(79.6)	47(20.4)	7.0(4.32,11.38)	3.72(2.07, 6.65)	
CPR received					
Yes	76(90.5)	8(9.5)	7.5(3.48, 16.15)	3.65(1.52, 8.78)	0.004*
No	152(55.9)	120(44.1)	1.0	1.0	
Obstetric complication during pregnancy					
Yes	62(88.6)	8(11.4)	5.6(2.59, 12.13)	1.43(0.57, 3.56)	0.440
No	166(58)	120(42)	1.0	1.0	
Pregnancy type					
Single	190(61.1)	121(38.9)	1.0	1.0	0.145
Multiple	38(84.4)	7(15.6)	3.46(1.45,7.99)	2.14(0.77, 5.97)	
Time of delivery					
Day time	108(56.8)	82(43.2)	1.0	1.0	0.352
Night time	120(72.3)	46(27.7)	1.98(1.26, 3.09)	1.32(0.73, 2.37)	

^*^ Significant at *p*-value ≤ 0.05

## Discussion

The prevalence of Neonatal hypothermia among newborn in this study was 64%. This was almost similar with a study conducted in Nigeria (62%) [12], in Bahir Dar, Ethiopia (67%) [22] and Gondar, Northwest Ethiopia (69.8%) [13]. And it was lower than a study conducted in Nepal (92.3%) [10], Zimbabwe (85%) [8] and Uganda (83%) [19]. But it was higher than a study conducted in South Africa (21%) [23], Bangladesh (34%) [9] and Pakistan (49.5%) [24]. This variation might be due to the difference in temperature measurement site, ecological, economic and cultural difference between the study areas.

There was high prevalence of hypothermia among out born delivery (65.9%); this might be due to lack of proper thermal care practice during inter-facility transportation. Neonates are transported from ward to ward or to other Hospital without proper wrapping. This finding was higher than a study done in Bangladesh which was 43% for out born and 22% for inborn but lower than Nigeria which was 90.9% for out born and 61.1% for inborn [9, 12, 23]. This might be due to the difference in inter-Hospital transport thermal care services, distance traveled to the hospital and economical difference.

This study revealed that Neonates with the age of 24 h old or less were 2 times more likely to have hypothermia than age greater than 24 h (AOR = 2.26, 95%CI: 1.27, 4.03). This could be due to the fact that newborns have no adequate adipose brown tissue and had no shivering thermogenesis so they are not capable for thermoregulation. This is similar to a study conducted in Bangladesh, (AOR = 2.23 95% CI: 1.22, 4.0) [9].

Preterm Neonates were 4.8 times more likely to have hypothermia when compared to term Neonates (AOR = 4.81, 95% CI: 2.67, 8.64). The possible reason might be preterm Neonates have immature and thin skin that increase heat loss through radiation, underdeveloped hypothalamic control, they lack efficient neural mechanisms for temperature control by shivering, have decreased glycogen stores, have decreased fat for insulation and have less brown adipose tissue, so they have decreased ability to regulate their body temperature, by producing heat through non - shivering thermogenesis [2, 25, 26]. This is almost similar to a study done in Pakistan in which preterm Neonates were 4 times more likely to develop hypothermia when compared to term newborn [24]. But it is higher than a study conducted in Iran in which preterm Neonates were 1.73 times more likely to be hypothermic than term one [27]. This variation might be due to the difference in the thermal care of preterm newborn, standard of delivery room and NICU.

Neonates who had no skin to skin contact with their mother immediately after delivery were 4.3 times more likely to develop hypothermia when compared with those who have skin to skin contact immediately after delivery (AOR = 4.39, 95% CI: 2.38, 8.11). The possible reason could be in the utero body temperature of the fetus is consistent with maternal temperature; Neonates who had skin to skin contact immediately after delivery with their mother gain heat through conduction which is consistent with their temperature in the womb during exposure of the newborn to extra uterine environment [28]. This finding is almost similar with a study conducted in Gondar, North west Ethiopia in which those who had no skin to skin contact were 3 times more likely to develop hypothermia [13]. Putting newborn together with the mother or kangaroo mother care is an important means of prevention of hypothermia [29].

Those Neonates who had no early initiation of breastfeeding within one hour after birth were 3.7 times more likely to be hypothermic when compared to those who had started breastfeeding within one hour after birth (AOR = 3.72, 95% CI: 2.07, 6.65). This might be due to the reason that breast milk is the source of energy or calories to produce heat for thermoregulation and they have no adequate adipose tissue for glucose breakdown which results in hypothermia [25]. And it is consistent with a study done in Nigeria but lower than a study done in Gondar, North west Ethiopia in which those who were delayed in initiation of breast feeding were 7.5 times more likely to be hypothermic [13, 18]. This difference in magnitude might be due to difference in study setup, knowledge of mothers on good positioning and attachment of breast feeding and difference in place of delivery.

Neonates who had resuscitation at birth were 3.6 times more likely to be hypothermic when compared to those who had no resuscitation (AOR = 3.65, 95% CI: 1.52, 8.78). This is due to the fact that Neonates who need resuscitation are those who had birth asphyxia; there is no enough oxygen which is needed for mitochondrial oxidation in the brown adipose tissue, for heat production. And during resuscitation at birth temperature control may not be properly taken care of; during emergency condition resuscitation may be done without wrapping the baby and in cold table. This finding is higher than study done in Bangladesh in which Neonates that had resuscitation were 2 times more likely to be hypothermic(AOR = 2.15, 95% CI:1.4–3.32) [9] and a study done in Iran in which those who had resuscitation at birth were almost 2 times more likely to be hypothermic (AOR =1.91, *p* value = 0.001) [27]. This variation may be due to the difference in thermal care practice during resuscitation, warm resuscitation or not and difference in time of resuscitation.

In bivariate analysis, low birth weight was statistically significant with the onset of hypothermia but in multiple logistic regression analysis it was not significant but there was a high prevalence of hypothermia among low birth weight neonates 127 (73.4%) compared with 101 (55.2%) normal birth weight. This is consistent with a study done in Pakistan 58.1%, Nigeria 89.1% and Gondar, Northwest Ethiopia 58 (89.2%) [13, 18, 24].

### Limitation of the study

Even though the study was conducted in multiple Hospitals, it was done with small sample size and it was conducted with short period of time or in one season so factors like climatic changes or seasonal variations were not addressed.

## Conclusions

The prevalence of Neonatal hypothermia among Neonates admitted to Neonatal Intensive Care Unit of Public hospitals in Addis Ababa was high 228 (64%). Preterm delivery, age of newborn ≤24 h, and absence of skin to skin contact with their mother immediately after delivery, delayed in early initiation of breastfeeding within one hour after birth and resuscitation at birth were factors that had significant association with Neonatal hypothermia. Therefore attention is needed for thermal care of preterm newborn and on the principle of WHO warm chain especially on early initiation of breast feeding, skin to skin contact and warm resuscitation. It is better to increase the practice of skin to skin contact immediately after delivery which is the effective warm chain principle especially in developing countries in which advanced warming instruments and incubators are not present.

## Additional file

Additional file 1: English version questionnaire, for the assessment of Neonatal Hypothermia and associated factors among Neonates admitted to Neonatal Intensive Care Unit of Public Hospitals in Addis Ababa, Ethiopia. (DOCX 23 kb)

## Abbreviations

^0^c: Degree centigrade
°F: Degree farhanite
AOR: Adjusted odds ratio
CI: Confidence interval
CPR: Cardio pulmonary resuscitation
ENBC: Essential newborn care
GA: Gestational age
MDG: Millennium development goal
NICU: Neonatal Intensive Care Unit
RR: Relative risk
SDG: Sustainable development goal
SPSS: Statistical Package for Social Sciences
WHO: World Health Organization
