# Supplementary material for: Neonatal hypothermia and associated factors among neonates admitted to neonatal intensive care unit of public hospitals in Addis Ababa, Ethiopia
Source: BMC Pediatr. 2018 Aug 4;18:263. doi: 10.1186/s12887-018-1238-0 (PMC6090740; doi:10.1186/s12887-018-1238-0)
Supplement: Supplementary file 1 — English version questionnaire, for the assessment of Neonatal Hypothermia and associated factors among Neonates admitted to Neonatal Intensive Care Unit of Public Hospitals in Addis Ababa, Ethiopia. (DOCX 23 kb) [file 12887_2018_1238_MOESM1_ESM.docx]

**English version questionnaire, for the assessment of Neonatal Hypothermia and associated factors among Neonates admitted to Neonatal Intensive Care Unit of Public Hospitals in Addis Ababa, Ethiopia.**

**General instruction**

1. For multiple choice questions chose one best answer
2. If your answer is not listed among alternatives, please tell your own answer for data collector.
3. If you have any question on the interview you can ask the data collector
4. If there is any problem during the study communicate with the supervisor

**Part I : Socio demographic characteristics**

| **No** | **Question** | **Response** |
| --- | --- | --- |
| 101 | Age of the mother | _____________ |
| 102 | Ethnicity | 1. Amhara 2. Tigre 3. Oromo 4. If other specify__________ |
| 103 | Religion | - 1. Orthodox   2. Muslim   3. Protestant   4. Catholic   5. If other specify……………. |
| 104 | Residence | 1. Urban 2. Rural |
| 105 | Educational status of the mother | 1. Unable to read and write 2. Primary school 3. Secondary school 4. Diploma and above |
| 106 | Occupation of the mother | 1. House wife 2. Government employ 3. Private business 4. Student 5. Farmer |
| 107 | Monthly income of the family | ……………… |
| 108 | Parity | 1. Primiparous 2. Multiparous |

**Part II: Hypothermia and Associated factor assessing question**

| 109 | Age of new born in hours | _____________ |
| --- | --- | --- |
| 110 | Sex of new born | 1. male 2. Female |
| 111 | Birth weight in gram | ………………… |
| 112 | Gestational age (GA) in weeks | ………………… |
| 113 | Axillary temperature of the new born At admission in ^o^c | ………………….. |
| 114 | Have you ever bathed your baby before 24 hours old? | 1. Yes 2. No |
| 115 | Have you provide skin to skin contact immediately after birth? | 1. Yes 2. No |
| 116 | Have you apply oil massage of the skin immediately after birth? | 1. Yes 2. No |
| 117 | Did the new born started breast feeding within one hour after birth? | 1. Yes 2. No |
| 118 | Did the new born received CPR during birth? | 1. Yes 2. No |
| 119 | Did you had obstetric complication during pregnancy? | 1. Yes 2. No |
| 120 | How was the pregnancy type? | 1. Single 2. Twine 3. Triple 4. Quadruple |
| 121 | What was the mode of delivery? | 1. Spontaneous Vaginal delivery 2. Instrumental 3. C/S |
| 122 | Where did you deliver? | 1. Inborn 2. Out born |
| 123 | If your delivery is out born in which setting you deliver? | 1. Other Hospitals 2. Health Centre 3. Private health facility 4. Traditional birth center 5. Homes |
| 124 | What was the time of delivery? | 1. Day time 2. Night time |
| 125 | How much was the room temperature of NICU in ^o^c | ……………………… |
| 126 | What is the Clinical diagnoses during admission | …………………. |
